# Development and validation of nomograms predicting postoperative survival in patients with chromophobe renal cell carcinoma

**DOI:** 10.3389/fonc.2022.982833

**Published:** 2022-11-14

**Authors:** Shuaishuai Li, Jiawei Zhu, Zhenwei He, Raj Ashok, Ning Xue, Zijie Liu, Li Ding, Haitao Zhu

**Affiliations:** ^1^ Department of Urology, The Affiliated Hospital of Xuzhou Medical University, Xuzhou, China; ^2^ Department of Neurosurgery, The Affiliated Hospital of Xuzhou Medical University, Xuzhou, China

**Keywords:** chromophobe renal cell carcinoma, nomogram, SEER, prognosis, overall survival, cancer-specific survival, validation

## Abstract

**Objective:**

The purpose of **o**ur study is to construct and validate nomograms that effectively predict postoperative overall survival and cancer-specific survival for patients with chromophobe renal cell carcinoma (chRCC).

**Method:**

Clinical, social, and pathological data from 6016 patients with chRCC collected from the SEER database were screened from 2004 to 2015. They were randomly assigned to a training cohort (n = 4212) and a validation cohort (n = 1804) at a 7:3 ratio. Cox regression and least absolute shrinkage and selection operator (LASSO) analyses were used to identify the prognostic factors affecting overall survival (OS) and cancer-specific survival (CSS) and establish nomograms. Their performance was validated internally and externally by calculating Harrell’s C-indexes, area under the curve (AUC), calibration, and decision curves. For external validation, samples from postoperative patients with chRCC at 3 independent centers in Xuzhou, China, were collected. Risk stratification models were built according to the total scores of each patient. Kaplan-Meier curves were generated for the low-risk, intermediate-risk, and high-risk groups to evaluate survival.

**Results:**

The C-indexes, AUC curves, and decision curves revealed the high ability of the nomograms in predicting OS and CSS, overall better than that of AJCC and TNM staging. Moreover, in internal and external validation, the calibration curves of 5-, 8-, and 10-year OS agreed with the actual survival. Kaplan-Meier curves indicated significant differences in survival rates among the 3 risk groups in OS or CSS.

**Conclusion:**

The nomograms showed favourable predictive power for OS and CSS. Thus, they should contribute to evaluating the prognosis of patients with chRCC. Furthermore, the risk stratification models established on the nomograms can guide the prognosis of patients and further treatment.

## Introduction

Chromophobe renal cell carcinoma (chRCC) is a subtype of renal cell carcinoma (RCC)and is its third most common subtype. It accounts for approximately 5% of all RCC cases, and this incidence is second only to papillary (15%) and clear cell renal carcinoma (70%-80%) (1). Histologically, chRCC consists of large polygonal cells with a clear/eosinophilic microreticular cytoplasm (2, 3). Its 5-year disease-free survival is significantly higher than that of clear cell, sarcomatoid, and papillary renal cell carcinomas (4). Although the prognosis of chRCC is generally better than that of other RCC subtypes, a 6%-7% probability of tumor progression and metastasis exists (5). For years, chRCC has received little attention because of its low incidence compared with other types of RCC. Moreover, tumor metastasis accounts for a large proportion of cancer-related deaths, causing factors that contribute to very different survival outcomes than those from existing studies (6, 7). Therefore, studying the factors affecting the prognosis of patients with chRCC to guide decision-making in the clinical management of the tumor, is essential.

The American Joint Committee on Cancer (AJCC) TNM staging system is the most widely recognized staging system for predicting the prognosis of patients with RCC (8) and has been used for decades. However, one pathological feature among patients with RCC often confers different prognoses. This effect may stem from individual differences in age, sex, race, and tumor size (9). Thus, another comprehensive, accurate tool is needed to individualize the assessment of prognosis in patients with chRCC.

Artificial intelligence is increasingly aiding healthcare. For example, a machine learning algorithm predicts the risk of developing the M1b stage of germ cell testicular cancer (10). Furthermore, a web-based model estimates the cancer-specific survival of elderly patients with clear cell RCC (11). Nomograms are statistics-based prediction tool that integrates key predictors and are widely used for risk quantification and prognostic assessment of multiple cancer types (12). Over the past 10 years, they have been well established in the RCC field, helping foresee the survival of patients. However, nomograms predicting the survival outcomes for patients with the chRCC subtype are lacking.

In this study, we aimed to construct nomograms that effectively predict postoperative overall survival (OS) and cancer-specific survival (CSS) for patients with chRCC. We compared their ability to predict survival with that of the AJCC and TNM staging. In addition, to test their performance, we did internal and external validation.

## Materials and methods

### Patient population

The Surveillance, Epidemiology and End Results (SEER) provides cancer statistics of patients registered in 17 regions, representing approximately 30% of the US population (13). Clinical, social, and pathological data of patients with chRCC (codes 8317/3, according to ICD-O-3) were gathered from the database. The SEER*Stat software (v 8.4.0) (account ID 12068, November 2021) was used to collect the data. The study involving human participants was conducted according to the Declaration of Helsinki and approved by the Ethics Committee of Affiliated Hospital of Xuzhou Medical University.

**Figure 1:**
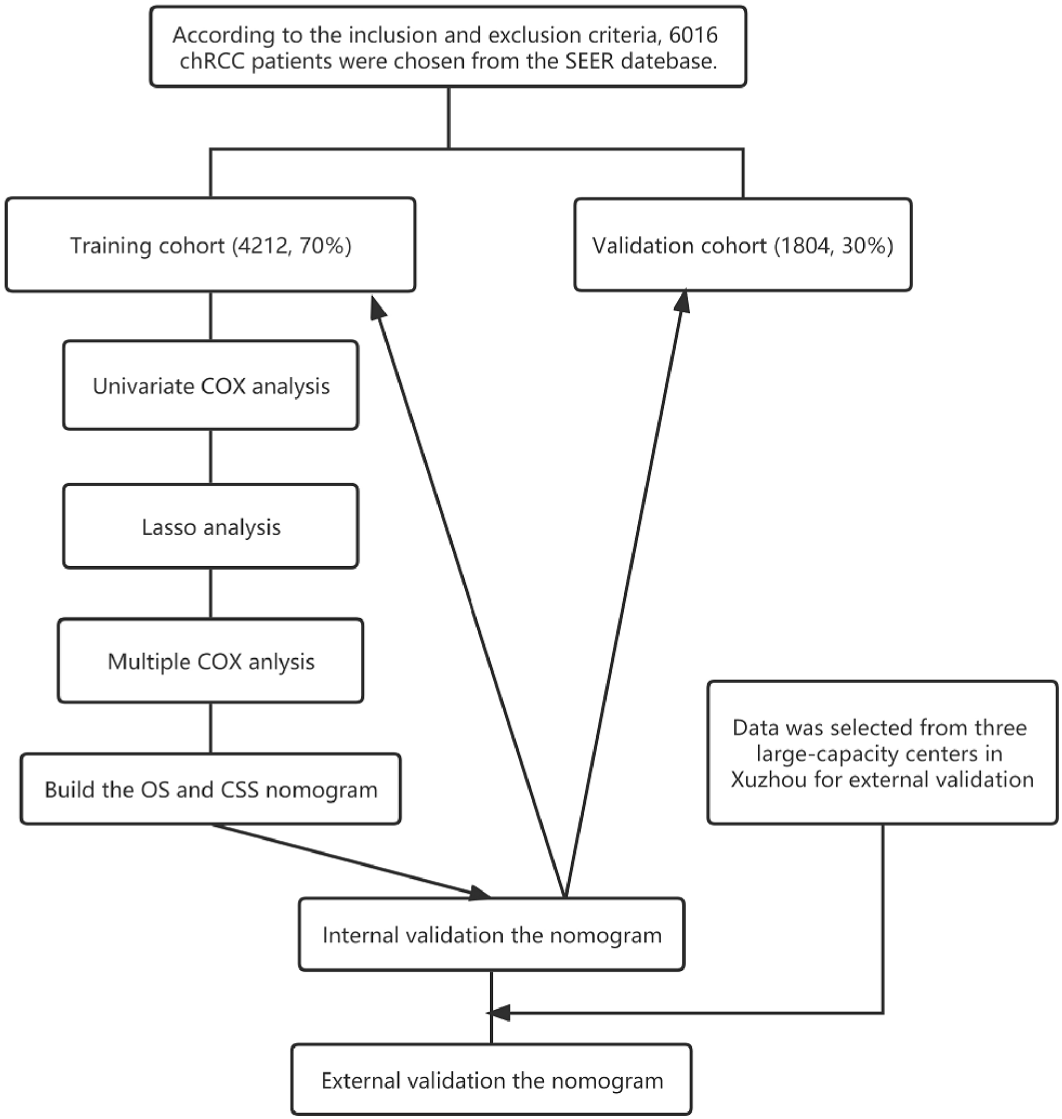
Flowchart of the study.

Patients with the following criteria were included in the study: (1) Primary ChRCC confirmed by postoperative pathological findings, (2) Surgically removed tumors. Those with the following criteria were excluded from the study: (1) Incomplete follow-up information, (2) Survival of 0 days. A total of 6016 patients, 5206 non-Hispanic and 810 Hispanic, were included. They were randomly assigned to a training cohort (to identify independent predictors of OS and CSS and build a nomogram survival prediction model) and a validation cohort (to internally verify our model) at a 7:3 ratio. In addition, the clinical data of 249 patients with pathologically confirmed chRCC (meeting the same criteria as above) were collected from 3 independent centers in Xuzhou, Jiangsu Province, China, from 2004 to 2015 for external verification of the nomogram function. The flow chart of this study is shown in **Figure 1**.

### Variables and follow-up information

The following variables were collected from the SEER database: clinical (sex, laterality, year of diagnosis, age at diagnosis, months from diagnosis to treatment, history of radiation and chemotherapy), social (race, marital status, median annual household income), and pathological (tumor grade, TNM and AJCC stages, tumor size). The TNM staging was performed using the sixth edition of the American Joint Council on Cancer (AJCC) TNM staging system. Tumor grading was divided into 4 histopathological types: well-differentiated (grade 1), moderately differentiated (grade 2), poorly differentiated (grade 3), and undifferentiated (grade 4). The last follow-up was in November 2021. The starting point of follow-up was the date of the chRCC diagnosis, while the endpoint was the date of death or the last follow-up.

### Statistical analysis

The annual diagnostic rate of chRCC from 2000 to 2019 was estimated and visualized using SEER*Stat (v 8.4.0) and Joinpoint (v 4.9.1.0) software. Diagrams illustrating the competing risks were generated with Yier oftware (v 2.0). Kaplan–Meier analysis was used to assess 5-, 8-, and 10-year OS and CSS.

X-tile software (Version 3.6.1, Yale University, New Haven, Connecticut, USA) (14) was used to determine the optimal cut-off points for age, year of diagnosis, months from diagnosis to treatment, and tumor size. The primary endpoints of the study were OS and CSS. Any two groups of data were compared with the *t* test or a nonparametric test.

The least absolute shrinkage and selection operator (LASSO) analysis was used to identify and select useful prediction factors, avoiding overfitting. The Cox proportional hazards regression was applied to determine the factors affecting the prognosis. Finally, the nomograms were constructed for OS and CSS based on the final risk factors.

The nomogram performance was subject to internal and external validation using Harrell’s C-index (the concordance index), AUC curves, and calibration, curves. Externally validated patient data were collected from 3 independent centers in Xuzhou, Jiangsu Province, China: Affiliated Hospital of Xuzhou Medical University, Xuzhou Central Hospital, and General Hospital of Xuzhou Mining Group. The calibration curves and Harrell’s C-index (the concordance index) were used to assess the nomogram performance.

Receiver operating characteristic (ROC) curve analysis is a statistical concept that cannot directly provide information on clinical value (15, 16). Therefore, decision curve analysis was used instead to evaluate the clinical applicability of the constructed nomograms. This analysis is increasingly used to evaluate the potential value of nomograms (15).

All statistical analyses were performed with R software (v 4.0.2) and SPSS (IBM, Armonk, NY, USA). Statistical significance was inferred when two-tailed *P* < 0.05.

## Results

### Patient characteristics

**Figure 2:**
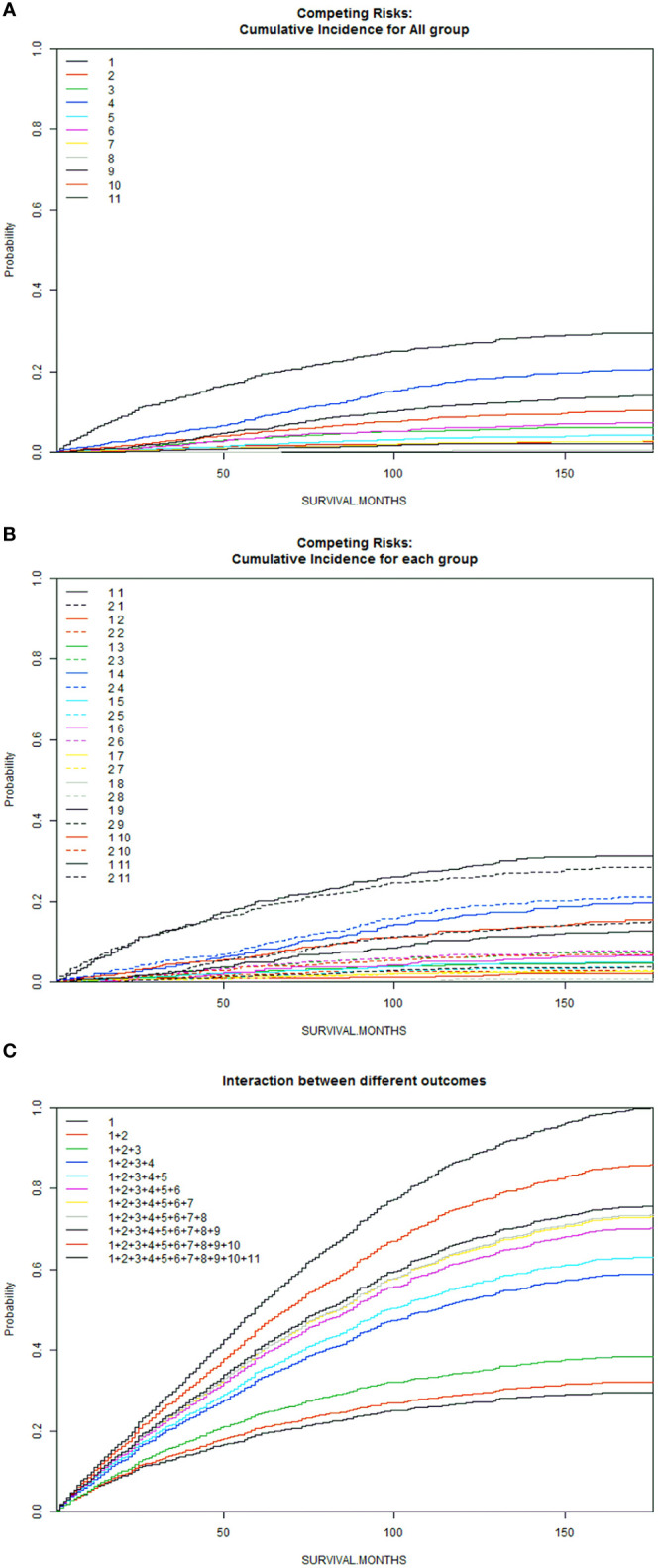
Competitive risk curves for causes of death in chRCC patients **(A)**: Cumulative Incidence for All Group; **(B)**: Cumulative Incidence for Each Group; **(C)**: Interaction Between Different Outcomes [Front (1: Female, 2: Male); Back (1: ChRCC; 2: Diseases of the blood system; 3: Digestive diseases; 4: Cardiovascular disease; 5: Nervous system and cerebrovascular diseases; 6: Respiratory diseases; 7: Diseases of endocrine system; 8: Other oncological diseases; 9: Urinary system and kidney diseases; 10. Other rare diseases (including suicide); 11: Unknown cause.)].

**Figure 3:**
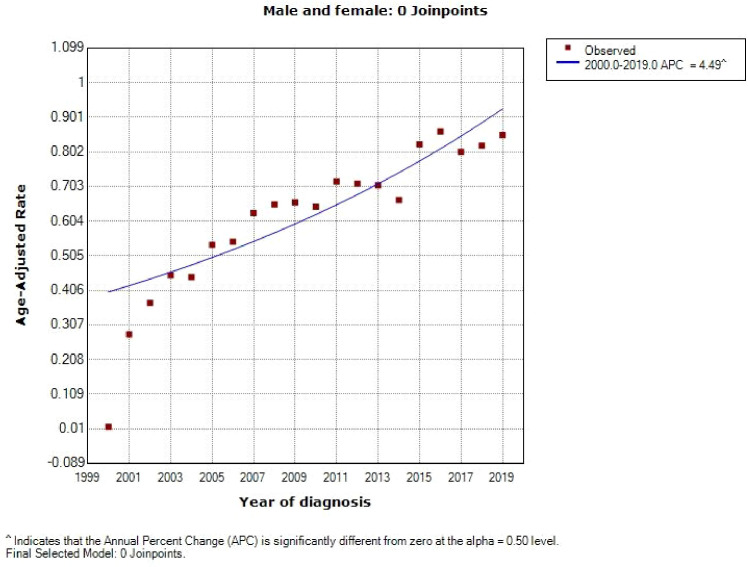
Diagnosis rate curves for chRCC patients from 2000 to 2019.

A total of 6016 patients with chRCC whose data were collected from the SEER database and who met the inclusion criteria were randomly assigned to a training cohort (n = 4212) and a validation cohort (n = 1804) at a 7:3 ratio. Their 5-, 8-, and 10-year OS and CSS are shown in **Table 1**, revealing a worse OS than CSS across all 3 temporal groups. We next estimated the competing risks, or death attributable to noncancerous causes, for the patients. As shown in **Figure 2**, noncancerous causes accounted for a substantial proportion of deaths among patients, of which cardiovascular diseases ranked first. Female patients also had a slightly higher CSS than males. The features of the patients are summarized in **Table 2**. Annual diagnostic rates of the patients from 2000 to 2019 (**Figure 3** **)** revealed that although the number of patients diagnosed with each consecutive year fluctuates, the number of patients with chRCC overall increases.

**Table 1:** Survivals of Chromophobe renal cell carcinoma(ChRCC), Based on SEER 2004–2015.

Year interval	Cancer-specific survival rate (% SE)	Overall survival rate (% SE)
5	95.8 (0.3)	89.0 (0.4)
8	93.9 (0.3)	81.6 (0.6)
10	92.8 (0.4)	76.5 (0.7)

SE indicates standard error of the mean.

**Table 2:** Patients’ demographics and clinicopathological characteristics.

Variables	No. of patients (%)N=6016	Training set, N=4212(70%)	Validation set, N=1804(30%)	P
Age of diagnosis	60 (50-70)	60 (50-70)	61 (50-70)	0.659
Race				0.377
White	4892 (81.3)	3414 (56.7)	1478 (24.6)	
Black	744 (12.3)	522 (8.7)	222 (3.7)	
Other	324 (5.3)	234 (3.9)	90 (1.5)	
Unknown	56 (0.9)	42 (0.7)	14 (0.2)	
Sex				0.403
Female	2637 (43.8)	1861 (30.9)	776 (12.9)	
Male	3379 (56.1)	2351 (39.1)	1028 (17.1)	
Marital status				0.004
Married	3788 (62.9)	2605 (43.3)	1183 (19.7)	
Single (Never Married)	892 (14.8)	630 (10.5)	262 (4.4)	
Other	1023 (17.0)	753 (12.5)	270 (4.5)	
Unknown	313 (5.2)	224 (3.7)	89 (1.5)	
Laterality				0.158
Left	2931 (48.7)	2027 (33.7)	904 (15.0)	
Right	3078 (51.1)	2180 (36.2)	898 (14.9)	
Bilateral	2 (0.1)	2 (0.1)	0 (0.0)	
Other	5 (0.1)	3 (0.1)	2 (0.1)	
Median annual family income, (median US dollars*)				0.189
<35,000	66 (1.0)	44 (0.7)	22 (0.4)	
35,000-75,000	3993 (66.3)	2777 (46.2)	1216 (20.2)	
>75,000	1957 (32.5)	1391 (23.1)	566 (9.4)	
Months from diagnosis to treatment	0 (0-1)	0 (0-1)	0 (0-1)	0.654
T				0.433
T1	4041 (67.1)	2818 (46.8)	1223 (20.3)	
T2	1082 (17.9)	758 (12.6)	324 (5.4)	
T3	828 (13.7)	588 (9.8)	240 (4.0)	
T4	18 (0.2)	15 (0.2)	3 (0.1)	
Tx	47 (0.7)	33 (0.5)	14 (0.2)	
N				0.244
N0	5842 (97.1)	4097 (68.1)	1745 (29.0)	
N1	47 (0.7)	33 (0.5)	14 (0.2)	
N2	34 (0.6)	25 (0.4)	9 (0.1)	
Nx	93 (1.5)	57 (0.9)	36 (0.6)	
M				0.835
M0	5893 (98.0)	4127 (68.6)	1766 (29.4)	
M1	83 (1.4)	54 (0.9)	29 (0.5)	
Mx	40 (0.7)	31 (0.5)	9 (0.1)	
AJCC Stage				0.502
I	3954 (65.7)	2756 (45.8)	1198 (19.9)	
II	1042 (17.3)	732 (12.2)	310 (5.2)	
III	768 (12.8)	556 (9.2)	212 (3.5)	
IV	121 (2.0)	85 (1.4)	36 (0.6)	
Unknown	131 (2.2)	83 (1.4)	48 (0.8)	
Grade				0.674
I	325 (5.4)	221 (3.7)	104 (1.7)	
II	2171 (36.1)	1522 (25.3)	649 (10.8)	
III	1227 (20.4)	858 (14.3)	369 (6.1)	
IV	245 (4.1)	173 (2.9)	72 (1.2)	
Unknown	2048 (34.0)	1438 (23.9)	610 (10.1)	
Tumor Size	45 (28-71)	45 (28-71)	43 (28-71)	0.479
Radiation				0.704
No	5989 (99.6)	4194 (69.7)	1795 (29.8)	
Yes	27 (0.4)	18 (0.3)	9 (0.1)	
Chemotherapy				0.819
No/Unknown	5926 (98.5)	4148 (68.9)	1778 (29.6)	
Yes	90 (1.5)	64 (1.1)	26 (0.4)	

"*" means in US dollars, not RMB.

### Construction of prognostic nomograms for overall survival and cancer-specific survival

**Figure 4:**
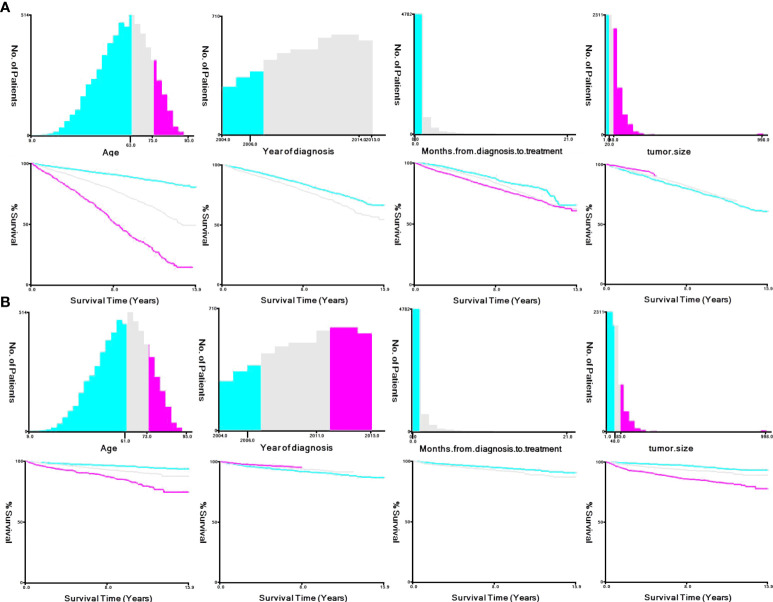
X-tile stratification. **(A)** Optimal cutoff points for age were 63 and 75 years in OS; P value of corresponding Kaplan–Meier curve was<0.05. Optimal cutoff point of year of diagnosis were 2006 and 2014; P value of corresponding Kaplan–Meier curve was<0.05 in OS. Optimal cutoff point of month from diagnosis to treatment was 1 in OS; P value of corresponding Kaplan–Meier curve was<0.05. Optimal cutoff point of tumor size were 20 mm and 48 mm in OS; P value of corresponding Kaplan–Meier curve was<0.05. **(B)** Optimal cutoff points for age were 61 and 73 years in CSS; P value of corresponding Kaplan–Meier curve was<0.05. Optimal cutoff point of year of diagnosis were 2006 and 2011; P value of corresponding Kaplan–Meier curve was<0.05 in CSS. Optimal cutoff point of month from diagnosis to treatment was 1 in CSS; P value of corresponding Kaplan–Meier curve was<0.05. Optimal cutoff point of tumor size were 48 mm and 85 mm in CSS; P value of corresponding Kaplan–Meier curve was<0.05.

We used X-tile software to stratify select clinical and pathological variables according to the cut-off criteria for predicting OS: age, ≤63 years, 64-75 years, ≥76 years; year of diagnosis, 2004-2006, 2007-2014, 2015; months from diagnosis to treatment, <1 month, ≥1 month; and tumor size, ≤20 mm, 21-48 mm, ≥49 mm. Those for predicting CSS were as follows: age, ≤61 years, 62-73 years, ≥74 years; year of diagnosis, 2004-2006, 2007-2011, 2012-2015; months from diagnosis to treatment, <1 month, ≥1 month; and tumor size, ≤48 mm, 49-85 mm, ≥46 mm (**Figure 4**).

**Figure 5:**
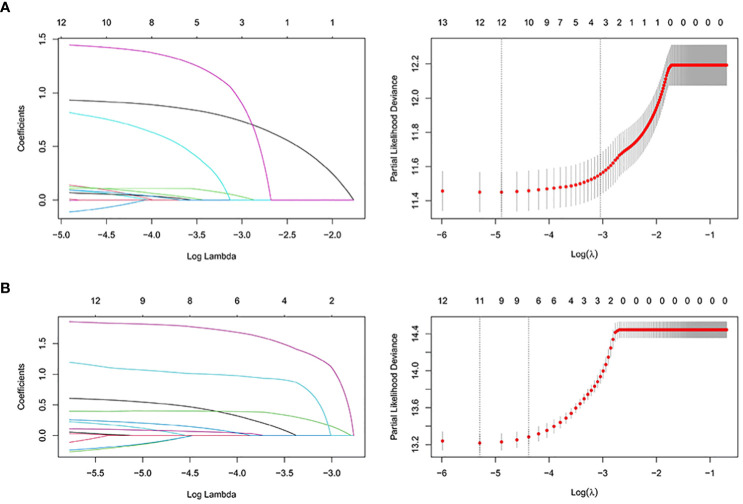
The LASSO regression used to select prognostic factors for OS and CSS. **(A)** LASSO coefficient profiles of variables for OS; LASSO analysis identified 12 variables for OS. **(B)** LASSO coefficient profiles of variables for CSS; LASSO analysis identified 12 variables for CSS. LASSO: least absolute shrinkage and selection operator.

We first analyzed the original 16 variables or predicting prognostic factors of OS with univariate Cox analysis and excluded those with *P* < 0.05 (**Table 3**). Subsequently, we performed the LASSO analysis to screen the remaining 14 variables (**Figure 5**). We also used the above principle for predicting the factors for CSS.

**Table 3:** Univariate and multivariate analyses of overall survival for training group.

Variables	Univariate analyseshazard ratios (95% CI)	P	Multivariate analyseshazard ratios (95% CI)	P
Year at diagnosis, Y				
2004-2006	Ref.		Ref.	
2007-2014	0.869 (0.743,1.016)	0.078	0.912 (0.777,1.071)	0.261
2015-2015	0.488 (0.324,0.734)	0.001	0.568 (0.376,0.858)	0.007
Age of diagnosis				
≤63	Ref.		Ref.	
64-75	2.760 (2.345,3.249)	0.000	2.983 (2.521,3.531)	0.000
≥76	7.320 (6.209,8.629)	0.000	7.055 (5.917,8.412)	0.000
Race				
White	Ref.			
Black	1.253 (1.043,1.505)	0.016		
Other	0.581 (0.401,0.842)	0.004		
Unknown	0.124 (0.017,0.880)	0.037		
Sex				
Female	Ref.		Ref.	
Male	1.187 (1.039,1.357)	0.012	1.295 (1.122,1.494)	0.000
Marital status				
Married	Ref.		Ref.	
Single	0.993 (0.808,1.221)	0.950	1.366 (1.106,1.686)	0.004
Other	1.934 (1.659,2.255)	0.000	1.582 (1.336,1.874)	0.000
Unknown	1.379 (1.028,1.851)	0.032	1.402 (1.038,1.893)	0.028
Laterality				
Left	Ref.			
Right	0.922 (0.809,1.051)	0.224		
Bilateral	5.340 (0.750,38.019)	0.094		
Unknow	1.042 (0.146,7.418)	0.976		
Median annual family income, (median US dollars*)				
<35,000	Ref.			
35,000-75,000	0.896 (0.480,1.673)	0.730		
>75,000	0.626 (0.333,1.178)	0.147		
Months from diagnosis to treatment				
<1	Ref.		Ref.	
≥1	1.292 (1.132,1.476)	0.000	1.129 (0.986,1.292)	0.079
T Stage				
T1	Ref.		Ref.	
T2	0.834 (0.688,1.010)	0.063	1.668 (0.746,3.728)	0.213
T3	1.965 (1.669,2.313)	0.000	1.889 (0.998,3.575)	0.051
T4	6.266 (3.352,11.713)	0.000	4.760 (1.937,11.696)	0.001
Tx	1.551 (0.803,2.997)	0.192	1.266 (0.413,3.882)	0.680
N Stage				
N0	Ref.		Ref.	
N1	4.925 (3.224,7.523)	0.000	2.136 (1.279,3.568)	0.004
N2	10.232 (6.618,15.819)	0.000	2.879 (1.622,5.111)	0.000
Nx	1.319 (0.804,2.163)	0.273	0.659 (0.245,1.771)	0.408
M Stage				
M0	Ref.			
M1	6.565 (4.813,8.953)	0.000		
Mx	0.682 (0.283,1.642)	0.393		
AJCC Stage				
I	Ref.		Ref.	
II	0.787 (0.645,0.962)	0.019	0.487 (0.212,1.117)	0.089
III	1.704 (1.430,2.031)	0.000	0.683 (0.356,1.314)	0.254
IV	7.060 (5.444,9.156)	0.000	1.669 (0.825,3.376)	0.154
Unknown	1.309 (0.846,2.024)	0.227	1.347 (0.511,3.551)	0.547
Grade				
I	Ref.		Ref.	
II	0.807 (0.604,1.078)	0.147	0.828 (0.618,1.109)	0.205
III	0.925 (0.684,1.252)	0.614	0.884 (0.650,1.203)	0.433
IV	1.637 (1.136,2.359)	0.008	1.405 (0.962,2.053)	0.079
Unknown	0.747 (0.556,1.005)	0.054	0.749 (0.555,1.010)	0.058
Tumor Size				
≤20	Ref.		Ref.	
21-48	1.304 (1.027,1.655)	0.029	1.109 (0.872,1.412)	0.398
≥49	1.569 (1.241,1.983)	0.000	1.479 (1.148,1.904)	0.002
Unknown	1.497 (0.654,3.428)	0.340	0.946 (0.233,3.843)	0.938
Radiation				
No	Ref.		Ref.	
Yes	11.512 (7.009,18.906)	0.000	2.430 (1.313,4.498)	0.005
Chemotherapy				
No/Unknown	Ref.		Ref.	
Yes	5.824 (4.268,7.948)	0.000	2.101 (1.338,3.298)	0.001

"*" means in US dollars, not RMB.

### Multivariate analysis

We next subject the 12 reviously screened variables to multivariate Cox analysis (**Table 3**). We identified those with a *P* < 0.05 as independent risk factors of OS: Age, Sex, Marital status, Year of diagnosis, Wait time, Tumor grade, TNM stage, AJCC stage, Tumor size, Radiation, and Chemotherapy.

We also performed a multivariate Cox analysis for CSS (**Table 4**) and determined variables with a *P* < 0.05 as its independent risk factors: Age group, Marital status, Median household income, Year of diagnosis, Wait time, Tumor grade, TNM stage, AJCC stage, Tumor size, Radiation, and Chemotherapy.

**Table 4:** Univariate and multivariateanalyses of cancer-specific survival for training group.

Variables	Univariate analyses hazard ratios (95% CI)	P	Multivariate analyseshazard ratios (95% CI)	P
Year at diagnosis, Y				
2004-2006	Ref.		Ref.	
2007-2011	0.733 (0.552,0.973)	0.032	0.797 (0.593,1.072)	0.133
2012-2015	0.559 (0.395,0.793)	0.001	0.633 (0.438,0.915)	0.015
Age of diagnosis				
≤61	Ref.		Ref.	
62-73	1.788 (1.344,2.380)	0.000	2.283 (1.690,3.085)	0.000
≥74	3.579 (2.685,4.771)	0.000	3.895 (2.824,5.373)	0.000
Race				
White	Ref.			
Black	1.136 (0.806,1.602)	0.468		
Other	0.973 (0.577,1.641)	0.918		
Unknown	0.000 (0.000,7.570E+74)	0.914		
Sex				
Female	Ref.			
Male	1.051 (0.829,1.333)	0.680		
Marital status				
Married	Ref.		Ref.	
Single	0.949 (0.662,1.361)	0.777	1.234 (0.849,1.796)	0.271
Other	1.445 (1.081,1.932)	0.013	1.225 (0.895,1.675)	0.204
Unknown	1.116 (0.646,1.926)	0.695	1.347 (0.768,2.364)	0.299
Laterality				
Left	Ref.			
Right	0.947 (0.748,1.199)	0.651		
Bilateral	14.373 (2.007,102.948)	0.008		
Unknown	3.634 (0.507,26.022)	0.199		
Median annual family income, (median US dollars*)				
<35,000	Ref.		Ref.	
35,000-75,000	0.399 (0.188,0.848)	0.017	0.362 (0.168,0.782)	0.010
>75,000	0.299 (0.138,0.649)	0.002	0.266 (0.121,0.584)	0.001
Months from diagnosis to treatment				
<1	Ref.		Ref.	
≥1	1.449 (1.144,1.834)	0.002	1.229 (0.958,1.577)	0.105
T Stage				
T1	Ref.		Ref.	
T2	1.446 (1.024,2.043)	0.036	1.081 (0.412,2.835)	0.875
T3	5.250 (4.034,6.833)	0.000	1.478 (0.690,3.168)	0.315
T4	26.037 (13.192,51.3890	0.000	3.703 (1.020,13.446)	0.047
Tx	2.491 (0.791,7.840)	0.119	1.501 (0.311,7.237)	0.613
N Stage				
N0	Ref.		Ref.	
N1	15.527 (9.654,24.112)	0.000	2.806 (1.534,5.133)	0.001
N2	27.236 (16.950,43.766)	0.000	3.160 (1.285,7.772)	0.012
Nx	2.107 (0.993,4.469)	0.052	1.485 (0.321,6.878)	0.613
M Stage				
M0	Ref.		Ref.	
M1	20.125 (14.323,28.277)	0.000	1.094 (0.359,3.340)	0.874
Mx	0.000 (0.000,5.998E+112)	0.939	0.000 (0.000,5.785E+92)	0.923
AJCC Stage				
I	Ref.		Ref.	
II	1.260 (0.858,1.851)	0.236	0.712 (0.258,1.967)	0.513
III	4.217 (3.137,5.667)	0.000	1.762 (0.803,3.869)	0.158
IV	31.762 (22.992,43.876)	0.000	4.869 (1.464,16.189)	0.010
Unknown	1.986 (0.872,4.526)	0.103	2.348 (0.425,12.967)	0.328
Grade				
I	Ref.			
II	0.847 (0.472,1.520)	0.578		
III	1.427 (0.792,2.572)	0.236		
IV	3.887 (2.066,7.312)	0.000		
Unknown	0.853 (0.472,1.541)	0.599		
Tumor Size				
≤48	Ref.		Ref.	
49-85	1.959 (1.457,2.632)	0.000	1.822 (1.326,2.502)	0.000
≥86	3.781 (2.848,5.021)	0.000	2.651 (1.763,3.986)	0.000
Unknown	0.955 (0.133,6.854)	0.963	0.444 (0.033,5.995)	0.541
Radiation				
No	Ref.		Ref.	
Yes	30.010 (17.751,50.734)	0.000	2.849 (1.302,6.234)	0.009
Chemotherapy				
No/Unknown	Ref.		Ref.	
Yes	15.272 (10.684,21.831)	0.000	2.187 (1.241,3.857)	0.007

"*" means in US dollars, not RMB.

### Nomogram construction

**Figure 6:**
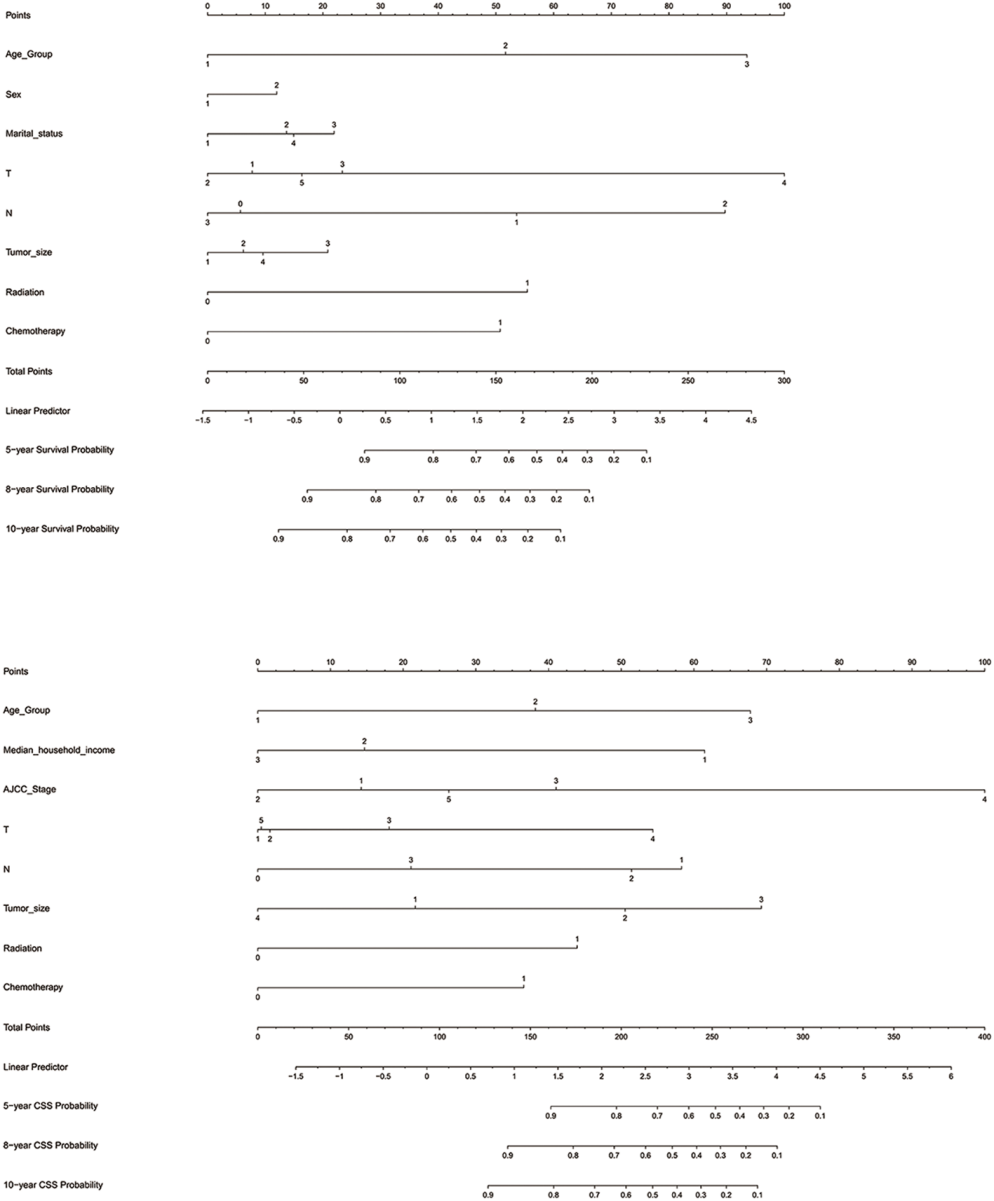
Nomograms for predicting 5-, 8-, and 10-year **(A)** OS and **(B)** CSS. OS, overall survival; CSS, cancer-specific survival; AJCC, American Joint Commission on Cancer; Marital status (Other): Divorced and Widowed.

We established nomograms that predict OS and CSS at 5, 8, and 10 years based on the independent prognostic factors (**Figure 6**). To obtain scores for each predictor, we projected different subtypes of each independent prognostic factor onto a score scale. The scores corresponding to each factor were then added to obtain an overall score. A scoring system allocates 0-100 points based on the contribution of each subgroup variable. The scores for all registry variables are summed to generate a total score for the underlying scale. We converted this score to predict the corresponding 5-, 8-, and 10-year OS and CSS. The higher the score of a variable, the greater its impact on prognosis.

The nomogram predicting OS of patients with chRCC revealed that the T stage (i.e., tumor invasion) was the most significant prognostic factor, followed by age and the N stage (i.e., lymph node involvement) (**Figure 6A**). Among the factors predicting CSS, the most intensive contributors to prognosis were AJCC stage, tumor size, and age. Furthermore, median household income, the N stage, and the T stage were moderate predictors of CSS (**Figure 6B**).

### Validation of nomogram performance

#### Internal validation

We first subject the nomograms predicting OS and CSS to internal validation with Harrell’s C-index. In the training cohort, the C-indexes of the nomogram for OS and CSS were 0.748 and 0.826, respectively. They were higher than AJCC stage (OS, 0.586; CSS, 0.720) and TNM stage (OS, 0.587; CSS, 0.726). In the validation cohort, the C-indexes of the nomogram for OS and CSS were 0.761 and 0.850, respectively, and higher than AJCC stage (OS, 0.572; CSS, 0.708) and TNM stage (OS, 0.572; CSS, 0.705).

**Figure 7:**
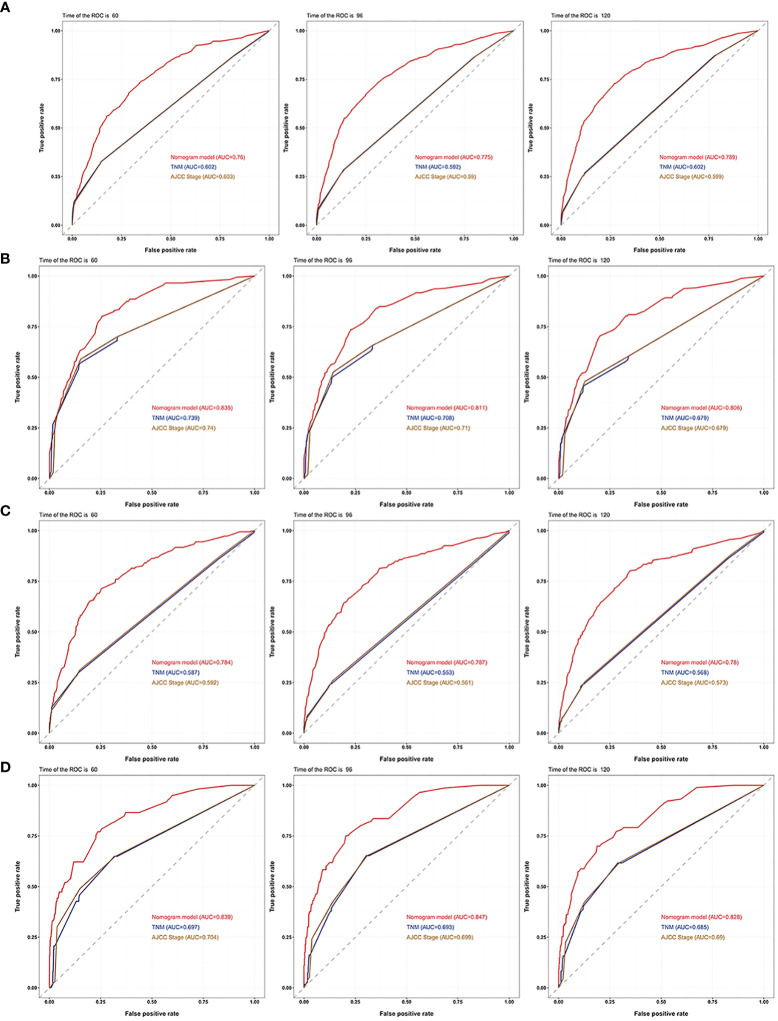
AUC curves of the nomogram, AJCC stage, and TNM stage for OS and CSS. AUC curves of the nomogram, AJCC stage, and TNM stage in prediction of prognosis at 5-, 8-, and 10-year point in the **(A)** training cohort for OS, **(B)** training cohort for CSS, **(C)** validation cohort for OS and **(D)** validation cohort for CSS.

The 5-, 8-, and 10-year area under the curve (AUC) curves of the nomogram for predicting OS in the training cohort were 0.760, 0.775, and 0.789, respectively. In addition, they were significantly higher than AJCC stage (0.603, 0.590, and 0.599) and TNM stage (0.602, 0.592, and 0.602) (**Figure 7A**). The AUC curves of the nomogram for predicting CSS in the training cohort were 0.835, 0.811, and 0.806, respectively. They were also higher than AJCC stage (0.740, 0.710, and 0.679) and TNM stage (0.739,0.708, and 0.679) (**Figure 7B**). We observed a similar trend for the 3 AUC curves for the nomograms in the validation cohort, which were also higher than that of the AJCC stage and TNM stage for either OS or CSS (**Figures 7C, D** **)**. Comparison of area under the curve (AUC) between the nomogram, TNM, and AJCC stages in chromophobe renal cell carcinoma patients are summarized in **Table 5**.

**Table 5:** Comparison of area under the curve (AUC) between the nomogram, TNM, and AJCC stages in chromophobe renal cell carcinoma patients.

Characteristics	AUC of training set			AUC of validation set		
OS	5-year	8-year	10-year	5-year	8-year	10-year
Nomogram	0.760	0.775	0.789	0.784	0.787	0.780
AJCC stage	0.603	0.590	0.599	0.592	0.561	0.573
TNM stage	0.602	0.592	0.602	0.587	0.553	0.568
CSS						
Nomogram	0.835	0.811	0.806	0.839	0.847	0.828
AJCC stage	0.740	0.710	0.679	0.704	0.699	0.690
TNM stage	0.739	0.708	0.679	0.697	0.693	0.685

**Figure 8:**
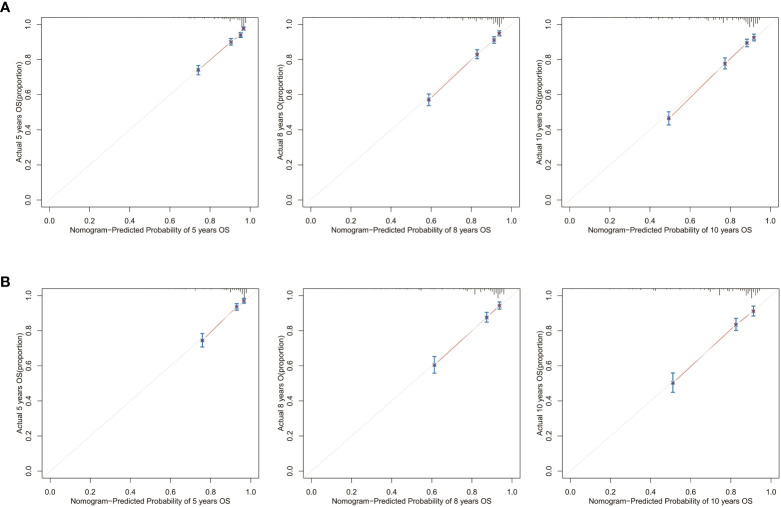
Calibration curves for the OS nomogram. 5-, 8-, and 10-year calibration curves for the OS nomogram in the **(A)** training cohort and **(B)** validation cohort.

**Figure 9:**
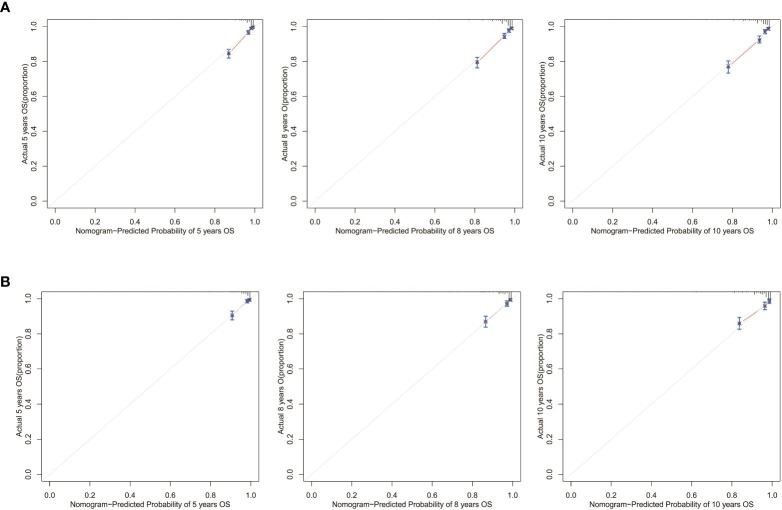
Calibration curves for the CSS nomogram. 5-, 8-, and 10-year calibration curves for the CSS nomogram in the **(A)** training cohort and **(B)** validation cohort.

Calibration curves for 5-, 8-, and 10-year OS approximate the gray line on the diagonal of the actual survival results of the training and validation cohort (**Figure 8**). Moreover, the survival rates, predicted with the nomograms, agree with the actual CSS rates in both cohorts (**Figure 9**). These results show that the actual survival of the training and verification cohort matches the predicted survival.

**Figure 10:**
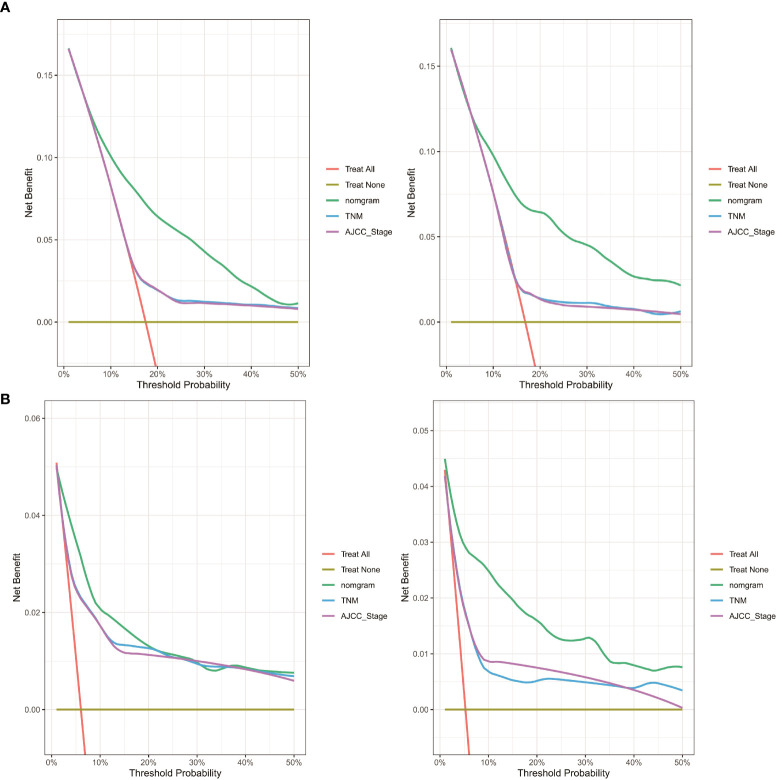
DCA curve of the nomogram, AJCC stage and TNM stage for **(A)** OS and **(B)** CSS in the training and validation cohort. DCA, decision curve analysis; AJCC, American Joint Commission on Cancer; OS, overall survival; CSS, cancer-specific survival.

Finally, as shown in **Figure 10**, decision curve analysis showed that the nomograms have a good predictive ability, better than AJCC or TNM staging.

#### External validation

**Figure 11:**
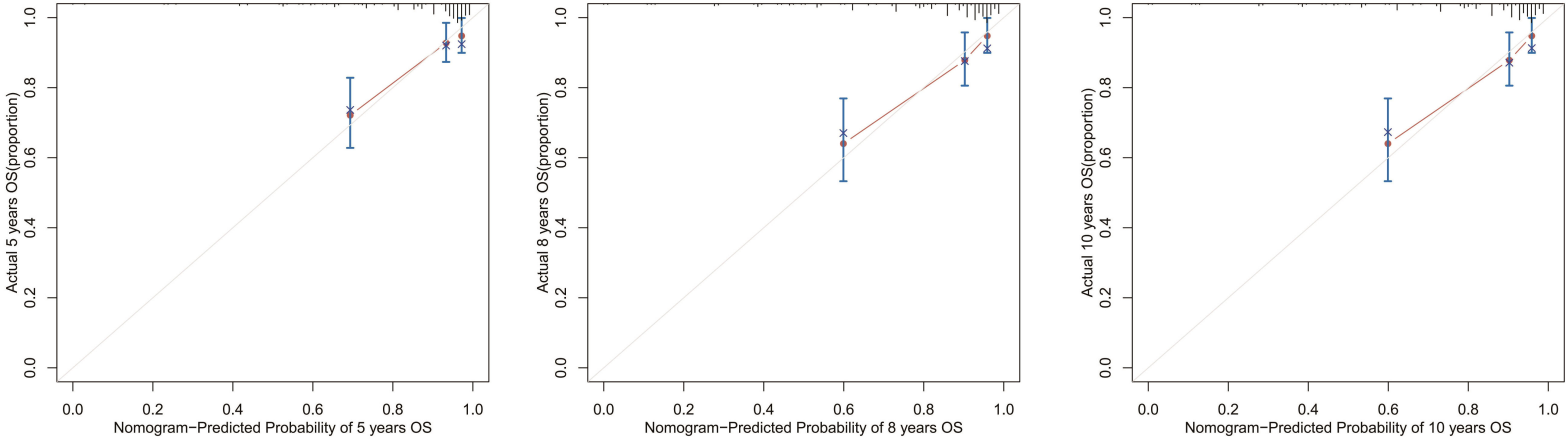
Calibration curves for the OS nomogram. 5-, 8-, and 10-year calibration curves for the OS nomogram in the external validation cohort.

Next, we collected data from 249 postoperative patients with chRCC from 3 independent centers in Xuzhou, China, for external validation. The calibration curves indicated that the 5-, 8-, and 10-year OS agree with the diagonal gray line of actual survival results (**Figure 11**).

#### Risk stratification model

**Figure 12:**
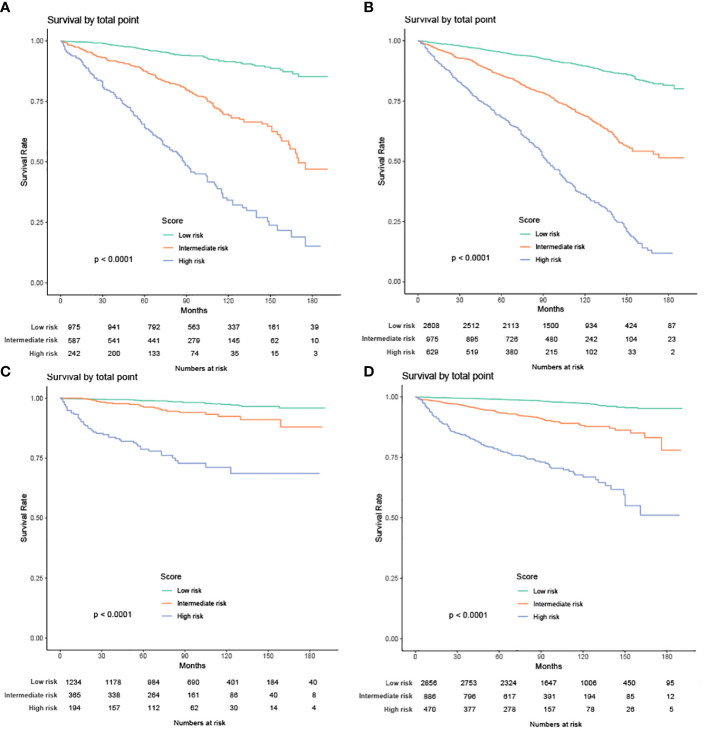
Kaplan-Meier curves of the low-, intermediate- and high-risk groups in training group and validation group for OS and CSS **(A)** Kaplan-Meier curves for OS in training group; **(B)** Kaplan-Meier curves for OS in validation group;**(C)** Kaplan-Meier curves for CSS in training group; **(D)** Kaplan-Meier curves for CSS in validation group).

We also built 2 risk stratification models based on the total score of each patient in the nomogram predicting OS or CSS. According to the risk stratification model, the patients were stratified into 3 groups: low-risk, intermediate-risk, or high-risk. Kaplan-Meier curves were plotted in both cohorts, demonstrating that this model can accurately distinguish survival in the 3 prognostic groups (**Figure 12**).

## Discussion

Large pale cells with reticulated cytoplasm, prominent cell membranes, and diffuse Hale’s iron colloid staining of the cytoplasm are the hallmarks of chRCC (17). Because of its low degree of malignancy, the survival rate of chRCC is similar to papillary renal cell carcinoma and higher than clear cell renal cell carcinoma (18, 19). Our results found that patients with chRCC have a high CSS, remaining as high as 92.8% at 10 years, consistent with previous studies (20). However, due to the high proportion of deaths from non-tumor causes (mainly cardiovascular diseases, and secondary blood and respiratory diseases), they have a much lower OS than CSS.Therefore, our external validation of the nomogram focused on the OS.

Due to the small sample size and low incidence of cancer-specific clinical events, studies on prognostic factors for chRCC are limited (21). Currently, no independent prognostic factor for chRCC is known. Therefore, we used the clinical data of 6016 patients with chRCC registered in the SEER database from 2004 to 2015 in this study, aiming to develop a model that can accurately predict the survival of patients in a large sample.

We observed that the rate of OS and CSS declines with age in patients older than 61 and 63, respectively. For CSS, the closer the date of birth to the current, the higher the survival. For OS, however, we showed the opposite effect. This phenomenon may arise from noncancerous diseases that also affect the OS of the patients, as we noted previously. Male divorced or widowed patients had poorer OS than females married or single. Fukushima et al. (22) demonstrated that female patients with clear cell renal cell carcinoma have a significantly better prognosis than males. A systematic review of the impact of marital status on cancer prognosis revealed that poor outcomes in divorced and widowed patients with cancer associate with poor financial status, poor mental health, and a lack of support network (23).For CSS, low household income was another factor conferring a poor prognosis. For both OS and CSS, tumor size had a negative correlation with prognosis. In addition, the history of chemotherapy and radiotherapy had a positive correlation with the prognosis of patients with metastatic or advanced chRCC.

The predictive factors in the OS nomogram were age, sex, marital status, T stage, N stage, tumor size, radiation, and chemotherapy. Those in the CSS nomogram also included median household income and AJCC stage. In both nomograms, T stage and AJCC stage were the biggest contributors to prognosis. The American Joint Committee on Cancer (AJCC) TNM staging system is the most extensively used and recognized for various cancers. Our results confirm its reliability in estimating outcomes in patients with chRCC (24). The Fuhrman grading system classifies RCC into 4 categories according to nuclear parameters. However, its applicability in grading chRCC is controversial (25). For instance, tumor cells of chRCC are usually defined as grade 3 due to their irregular nuclei and varying nuclear size (26). Paner et al. proposed an alternative 3-tiered chromophobe tumor grade (CTG) system to improve the classification of chRCC (6). We found that undifferentiated tumors are an independent factor in patient survival in univariate analysis. However, while screening variables for inclusion in the nomogram, we finally screened the Fuhrman grading system out.

In this study, we used social, clinical, and pathological information that is publicly available in the SEER database to construct nomograms. By observing AUCs over time, we concluded that the nomograms have a significant advantage over AJCC and TNM stages in validation and training cohorts. Calibration curve analysis also supports that nomograms perform well in predicting OS and CSS in the cohorts. Furthermore, the C-indexes and decision curves imply the nomograms have higher predictive accuracy than AJCC and TNM stages. We also performed external validation of the nomograms on patients admitted to 3 medical centers in Xuzhou, Jiangsu Province, China. The purpose of external validation was to demonstrate the generalization ability of the model, that is, its ability to predict datasets other than the modeled data. The abscissa of the calibration curve is the predicted risk, while the ordinate is the observed actual risk, ranging from 0 to 1, which can be understood as the event rate (percentage). The diagonal dotted line is the reference line, or where the predicted value equals the observed. The red line is the curve fitting line, and the colored part is the 95% CI. If the predicted value equals the observed, the red line exactly coincides with the reference line. If the predicted value is greater than the observed (the risk is overestimated), the red line shifts below the reference line. Finally, if the predicted value is less than the observed (the risk is underestimated) the black line shifts above the reference line. The closer the predictive calibration curve is to the standard curve, the better the predictive ability of the nomogram. The reason for external validation is that overfitting may occur during the modeling process. Because of overfitting, the model works well for the modeling dataset (experimental set) but not for other datasets (testing sets) and has little value for the researcher. External verification is also performed by calibrating the curve to further judge the predictive ability of the nomogram. The closer the predictive calibration curve is to the standard curve, the better the predictive ability of the nomogram. The results of our calibration curve analysis indicate the predicted and actual survival are consistent and demonstrate the generalizability of the nomogram. To our knowledge, few studies have used external validation of nomograms, underscoring the relevance of our work.

Nomograms help determine the prognosis of patients. According to the various influence factors of nomogram contribution degree of outcome variables (the size of the regression coefficient), for each level of each value of factors affecting the assigned points, then the scores to get the total score, and finally by the total score and event probability function conversion relationship between predicted values and the individual event is calculated. The higher the predicted value, the higher the risk level. According to the risk stratification model, we stratified all patients into 3 grades: low-risk, medium-risk, and high-risk. Patients in the low-risk group had a good prognosis, while those in the high-risk had a poor prognosis and needed further treatment after surgery. Finally, patients in the intermediate-risk group had an intermediate prognosis.

Our study has some limitations. First, it is a retrospective study and is prone to selection bias. Second, the SEER database lacks valuable information that may affect the survival of patients, such as surgical methods, postoperative complications, other diseases, and laboratory test indicators. It also lacks specific data on radiotherapy and chemotherapy of patients.

## Conclusions

We constructed nomograms and risk stratification models to predict individual survival in patients with chRCC. These models should help clinicians identify high-risk patients and provide more personalized treatment for patients with different prognoses.

## Data availability statement

The raw data supporting the conclusions of this article will be made available by the authors, without undue reservation.

## Ethics statement

Written informed consent was obtained from the individual(s) for the publication of any potentially identifiable images or data included in this article.

## Author contributions

SL, HZ and ZH conceived the study, participated in its design, collected the data, performed the statistical analysis, and drafted the manuscript. JZ, RA and NX helped to collected the data and performed the statistical analysis. LD and ZL helped to collected the data. We have agreed to be accountable for all aspects of the work in ensuring that questions related to the accuracy or integrity of any part of the work are appropriately investigated and resolved. All authors contributed to the article and approved the submitted version.

## Conflict of interest

The authors declare that the research was conducted in the absence of any commercial or financial relationships that could be construed as a potential conflict of interest.

## Publisher’s note

All claims expressed in this article are solely those of the authors and do not necessarily represent those of their affiliated organizations, or those of the publisher, the editors and the reviewers. Any product that may be evaluated in this article, or claim that may be made by its manufacturer, is not guaranteed or endorsed by the publisher.
